# Sol–Gel Co-Precipitation Synthesis, Anticoagulant and Anti-Platelet Activities of Copper-Doped Nickel Manganite Nanoparticles

**DOI:** 10.3390/gels7040269

**Published:** 2021-12-16

**Authors:** Shashidharagowda H., Shridhar Mathad, Shridhar Malladi, Vinod Gubbiveeranna, Kusuma C. G., Nagaraju S., Arun Y. Patil, Anish Khan, Abdul Rub Malik, Abdullah M. Asiri, Naved Azum

**Affiliations:** 1 Department of Physics, Tontadarya College of Engineering, Gadag 582101, India; hsgowdra@gmail.com; 2 Department of Engineering Physics, KLE Institute of Technology, Hubballi 580027, India; 3 Department of Chemistry, KLS Gogte Institute of Technology, Belagavi 590008, India; shridhar.malladi@gmail.com; 4 Department of Studies & Research in Biochemistry, Tumkur University, Tumkur 572101, India; vgubbiveeranna@gmail.com (V.G.); cgkusuma@gmail.com (K.C.G.); nagarajubiochem@gmail.com (N.S.); 5 School of Mechanical Engineering, KLE Technological University, Hubballi 580031, India; patilarun7@gmail.com; 6 Center of Excellence for Advanced Materials Research, King Abdulaziz University, Jeddah 21589, Saudi Arabia; anishkhan97@gmail.com (A.K.); malikrub@gmail.com (A.R.M.); aasiri2@gmail.com (A.M.A.); 7 Chemistry Department, Faculty of Science, King Abdulaziz University, Jeddah 21589, Saudi Arabia

**Keywords:** Cu-Ni manganites, anticoagulant, antiplatelet

## Abstract

Copper-substituted nickel manganites Ni_(1−x)_Cu_x_Mn_2_O_4_ (Ni-TCE-NPs) were produced by co-precipitation route (sol–gel) at room temperature. Ni_(1−x)_CuxMn_2_O_4_-Bio (NCB) NPs were studied by powder X-ray diffraction technique, scanning electron microscopy and Raman spectroscopy. XRD spectra authenticated the copper-doped nickel manganites’ formation with particle size 23–28 nm. A significant decrease in the lattice parameter confirmed the doping of copper ions into the nickel manganites. Microscopy (SEM) was used to estimate the grain size, shape and uniformity, revealing the non-uniform agglomerated polygon and plate-like microstructure. The NCB-NPs showed anticoagulant activity by enhancing the coagulation time of citrated plasma of human beings. NCB-NPs with x = 0.35 and 0.45 have increased clotting time from control 133 ± 4 s to 401 ± 7 s and 3554 ± 80 s, respectively, and others around 134 s. Additionally NCB-NPs with x = 0.35, 0.45 inhibited the platelet aggregation by 80% and 92%, while remaining inhibited with only 30%. NCB-NPs did not show hemolytic activity in RBC cells intimate its non-toxic nature. Finally, NCB-NPs were non-toxic and known to exhibit anti-blood-clotting and antiplatelet activities, which can be used in the field of biomedical applications, especially as antithrombotic agents.

## 1. Introduction

Magnetic substances have nano-technological applications in areas such as analytical chemistry, biosensing and nano-medicine. These magnetic nanoparticles (MNPs) are eco-friendlier and more compatible with the applications in the field of pharmacy and biomedicine [1,2]. Metallic nanoparticles are often modified with numerous chemical functional groups that permit them to conjugate with antibodies and medicines. Nickel nanoparticles have shown exceptional electrochemical and super-magnetic properties [3]. Engineered NPs with paramagnetic and super-magnetic properties showed profound potential in cancer recognition, drug delivery, gene delivery systems and molecular imaging [4,5,6,7]. Engineered nanoparticles are used as antithrombotic agents with potential anticoagulant and antiplatelet activities. Limited studies have been conducted on the NPs by using as anticoagulant and antiplatelet agents.

The thrombotic disorder is the major cause of death in cardiovascular disorders, directly resulting from pathological thrombogenesis or thrombus-exuviation-induced vascular embolism. Hyperactivation of blood coagulation factors in the coagulation cascade triggers downstream thrombin activation and platelet aggregation. The process culminates in the formation of insoluble fibrin, along with cellular components, eventually leading to the formation of thrombus. However, the main concern of NPs is their interaction with blood components. There is a potential in the study of NPs’ interaction with the components of the blood clotting system that may be an important part of their safety profile. Blood coagulation may be a tightly regulated mechanism that plays an important role in maintaining the fluidity of the blood and in arresting bleeding during injury. Several factors alter the coagulation mechanism, leading to the unnatural formation of blood clots in arteries and veins that may contribute to cardio/cerebrovascular disorders. Anticoagulant and antiplatelet agents prevent or delay the formation of thrombi. There are many challenges in clinical usage of anticoagulant drugs, such as short half-life in the plasma, repeated administration and high cost. Anti-blood-clotting nanoparticles (NPs) can help to reduce dosage and side effects and increase efficacy. The physical and chemical properties of the NPs’ surface show various types of interactions with the proteins that may lead to changes in protein conformation, activation or inhibition of blood-coagulation factors.

Manganese-based transition-metal oxides are extensively used to control, monitor and compensate the temperature in household appliances, manufacturing units, health sector, biomedicine, aerospace, cryogenics and automotive factories [8]. The literature reports that concern copper-doped nickel ferrites, with larger saturation–magnetization, electrical resistivity, magnetic permeability and moderate usage for biomedical applications [9] inspired us to perform the biological study on copper-doped nickel manganites. These manganites having a general formula AB_2_O_4_, in which cations reside at both tetrahedral (A-sites) and octahedral (B sites) showed good NTC thermistor properties, dielectric capabilities, magnetic nature and ease of production, are promising materials for biological investigation [10]. Quite a little information is available from the literature regarding anticoagulant and antiplatelet activities of copper-doped nickel manganite nanoparticles. Thus, in our work, we describe the synthesis and characterization of Ni_(1−x)_Cu_x_Mn_2_O_4_ (NCB-NPs), along with their effect on plasma coagulation and platelet aggregation.

## 2. Results and Discussion

Nanotechnology has been used in multiple applications in the field of therapeutics, such as early diagnosis, imaging and medicine-delivery systems [11]. There is a significant interest in the scientific society in further culturing the potential medicinal applications of NPs. In this view, the NPs–blood interaction is inevitable, and this study is about the effect of NCB-NPs on blood-clotting cascade and platelet aggregation.

### 2.1. Structural Studies

**Figure 1:**
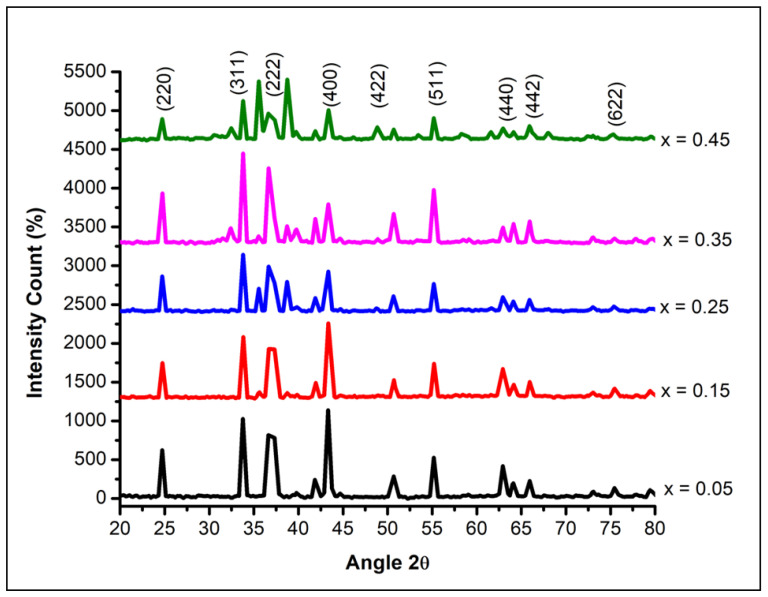
X-ray diffraction patterns of Ni_(1−x)_Cu_x_Mn_2_O_4_ with x = 0.05, 0.15, 0.25, 0.35, 0.45.

The X-ray diffraction spectra of NCB-NPs (x = 0.05, 0.15, 0.25, 0.35, 0.45) samples are presented in Figure 1. The X-ray diffraction patterns reveal a spinel cubic system, as their diffraction peaks and intensities matched with the XRD spectrum of cubic spinel NiMn_2_O_4_ (space group: Fd_3_m) with JCPDC card #00-084-0542. 

From the X-ray diffraction pattern, lattice parameter *a* is determined by using the following equation:

(1) $$a=d\times \sqrt{\left(h^{2}+k^{2}+l^{2}\right)}$$

where *d* is the distance between consecutive planes, and (*h k l*) are Miller indices of crystallographic planes. The value of lattice constant calculated ranges from 8.52 to 8.38 Å. Crystalline size, *D*, was determined with the help of the Debye–Scherer equation [12].

*D* = 0.9*λ*/*βcosθ* (2)

where *λ* is the wavelength of X radiations, *β* is the full width half maximum (FWHM) of diffraction peak and *θ* is Bragg’s angle. As XRD peaks of the synthesized sample are sharper and more intense, having a larger grain size of 3 μm, which will have higher electrical conductivity due to lower grain boundary resistance in accordance with Scherer expression. It was found from the below Table 1 that the lattice constant of NCB-NPs (x = 0.05, 0.15, 0.25, 0.35, 0.45) increased as the copper content increased because the ionic radius of copper (0.73 Å) is larger than nickel (0.69 Å) [13]. The estimated value of the mean crystalline size of the samples ranges from 24.15 to 27.15 nm. Even though all of the samples were synthesized under identical conditions, the particle size was dissimilar for all copper concentrations, and this may be due to preparation condition, which favors a change in particle size. 

### 2.2. Morphological Studies

**Figure 2:**
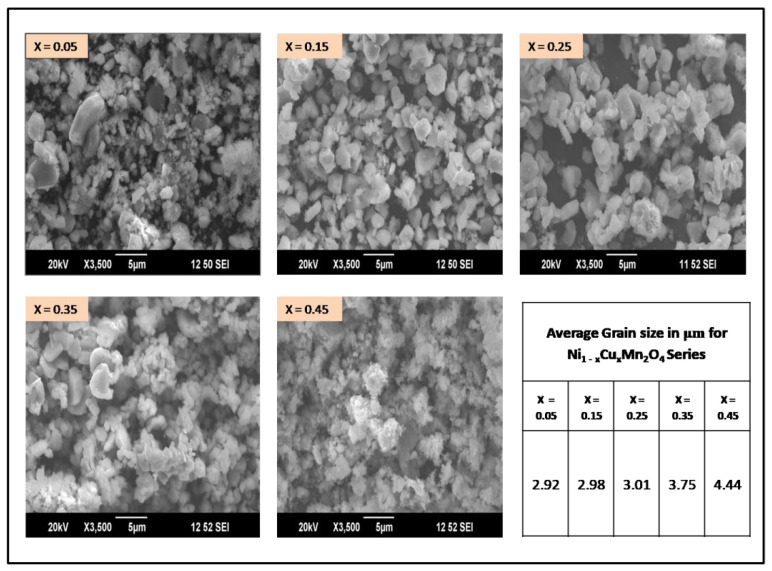
Scanning electron microscope images of Ni_(1−x)_Cu_x_Mn_2_O_4_ (x = 0.05, 0.15, 0.25, 0.35, 0.45).

The surface morphology details were obtained by analyzing the SEM images of prepared copper-doped nickel manganites, as presented in Figure 2, which depicts agglomerated non-uniform polygon and plate-like grains. The grain sizes of the samples were measured to be 2.5 to 4.5 μm, using IMAGEJ. The grain size gradually increases with copper, because of different atomic radii of nickel (0.69 Å) and copper (0.73 Å), which turns the grain texture into an agglomerated polyhedral plate-like microstructure with larger homogeneity.

### 2.3. Raman Studies

**Figure 3:**
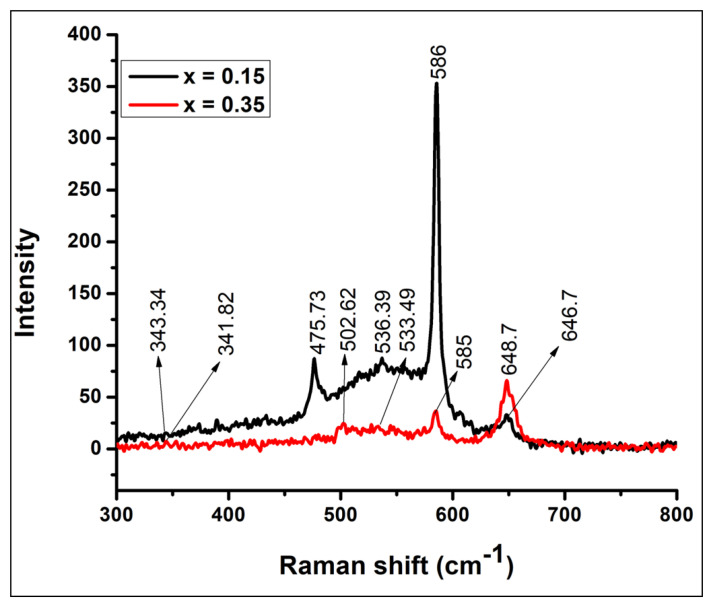
Raman spectrum of Ni_(1−x)_Cu_x_Mn_2_O_4_ with x = 0.15, 0.35.

**Figure 4:**
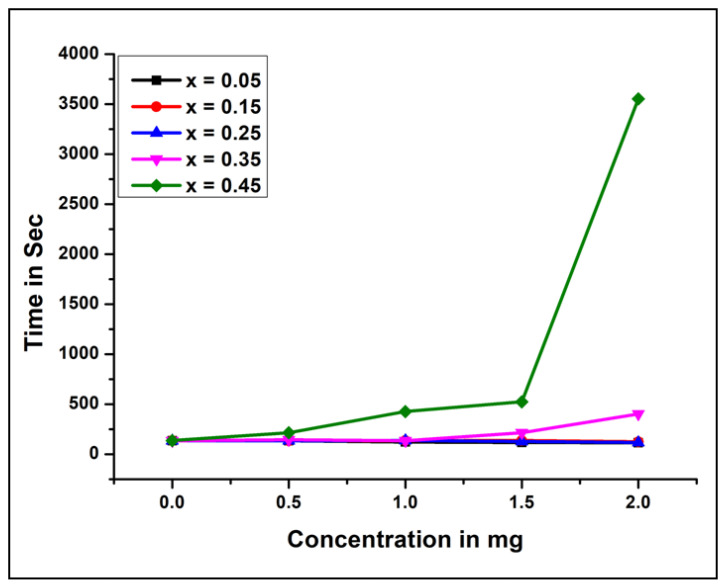
Coagulation time and concentration of Ni_(1−x)_Cu_x_Mn_2_O_4_ with (x = 0.05, 0.15, 0.25, 0.35, 0.45).

The Raman spectra of NCB-NPs (x = 0.15 and x = 0.35) with wavenumbers from 300 to 800 cm^−1^ are presented in Figure 3. As the spinel sample has a cubic system belonging to the symmetry group *O^7^_h_ (Fd-3m)*, it produces five Raman-sensitive states (A_1g_ + E_g_ + 3F_2g_) [14]. Figure 4 consists of broad bands at 480, 530 and 646 and a strong band at 585 cm^−1^. The wide peak at 646 cm^−1^ is attributed to A_1g_-sensitive states, which evolved due to symmetric expanding vibrations of Mn–O in MnO_6_ octahedral position. The remaining three peaks are attributed to F_2g_-sensitive states corresponding to Ni–O expanding vibration in both octahedral and tetrahedral positions [15]. The intensity of the peak at 586 (Ni–O bond) decreases, and that of 646 (Mn–O) increases, possibly due to decrement in Ni content and lower ionic radius of Mn (0.66 Å) compared to Ni (0.78 Å). According to Malavasi et al., the wave numbers of A_1g_ mode are inversely proportional to the lattice parameter of the spinel; even the shift in wavenumber of the Raman-active mode is only around 1%, which is evident from Figure 4 [16].

### 2.4. Anticoagulant and Antiplatelet Analysis

#### 2.4.1. Human Plasma Clotting Time

Hemostasis is a complex physiological process that drives immediate response upon blood vessel injury. Blood coagulation follows two pathways: the contact activation tract (intrinsic pathway) and the tissue factor tract (extrinsic pathway). These tracts independently culminate at a common tract that is the conversion of prothrombin (factor II) to thrombin (factor IIa). Thrombin is a serine protease that hydrolyzed soluble fibrinogen to form insoluble fibrin monomers. Later, fibrin monomer polymerizes, along with other cellular components, to form a fibrin clot that helps to plug damaged blood vessels, arresting the bleeding [17,18,19]. Meanwhile, impairment of homeostasis leads to the hyperactivation of the coagulation cascade factors, thus leading to thrombotic disorders [20,21,22,23]. Therefore, thrombosis is the pathological phenomenon involved in the production of an unnatural clot in the arteries and veins during surgical procedures, thus increasing the rate of mortality and morbidity [24].

#### 2.4.2. Citrated Human Plasma Coagulation Time

**Figure 5:**
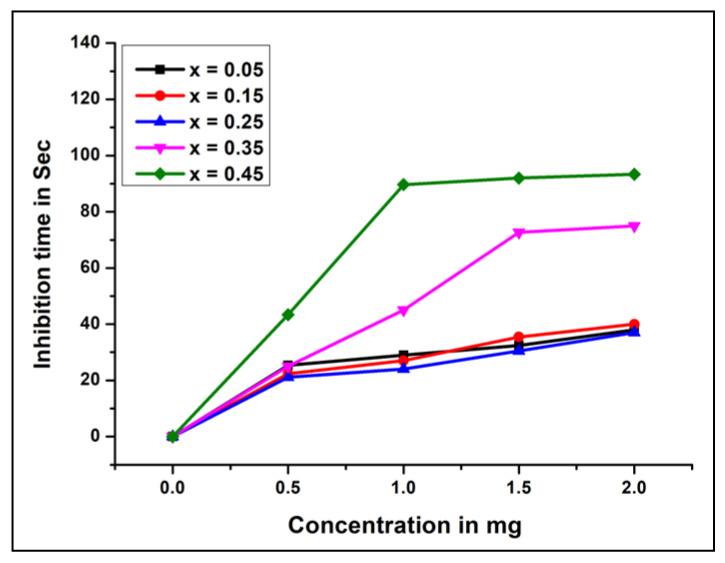
Inhibition time V/s concentration of Ni_(1−x)_Cu_x_Mn_2_O_4_ (x = 0.05, 0.15, 0.25, 0.35, 0.45).

Different concentrations of NCB-NPs were pre-cultured with 0.2 mL of citrated human plasma PRP/PPP, taking the help of 20 μL of 10m MTris-HCl buffer (pH 7.4) for 1 min at 37 °C. Then 20 μL of 0.25 M CaCl_2_ was put into the pre-cultured mixture, and the coagulation time was recorded. The plot of coagulation time and concentration of NCB-NPs is as shown in Figure 5. All results are mean ± SD of three individual experiments.

To evaluate the effect of NCB-NPs in coagulated plasma, the clotting time of citrated plasma of human beings was examined by using plasma rich in platelets. NCB-NPs showed the anticoagulant effect by increasing the plasma coagulation time of PRP from control 145 to 400 s and from 145 to 3000 s for NCB-NPs with x = 0.35 and 0.45, respectively, while they showed constant plasma coagulation time of PRP around 134 s only. The highest concentration used in the two cases was confirmed to be 2 mg. There is a need to synthesize NPs, which have anticoagulant properties that can be used to coat surgical instruments. Very few studies have reported the anticoagulant and thrombolytic effects of NPs. The NCB-NPs have shown significant anticoagulant effects.

#### 2.4.3. Antiplatelet Aggregation Property

The platelets play a significant role in the maintenance of hemostasis and thrombus formation. Platelets are the cellular components of blood clotting that originated from megakaryocytes in the bone marrow. Platelets consist of a peripheral zone with glycoprotein receptors; contractive microtubules; and organelles with alpha granules, dense granules and lysosomes. The activation of platelets is induced by binding of surface receptors to fibrinogen, thromboxane A2, thrombin, ADP, adrenaline, collagen and vWF [25]. NPs generally developed for the therapeutic purpose usually aims to treat injured vascular sites to enhance platelet aggregation [26]. While the anti-platelet aggregation has also a significant concern in the field of nano-medicine and might lead to the synthesis of engineered NPs for the clinical setting. There are reports of NPs inhibiting platelet aggregation, such as Ag NPs, Au NPs and polysorbate/PEG/anionic NPs [27].

#### 2.4.4. Antiplatelet Aggregation Effect of NCB-NPs with the Usage of ADP as an Agonist

**Figure 6:**
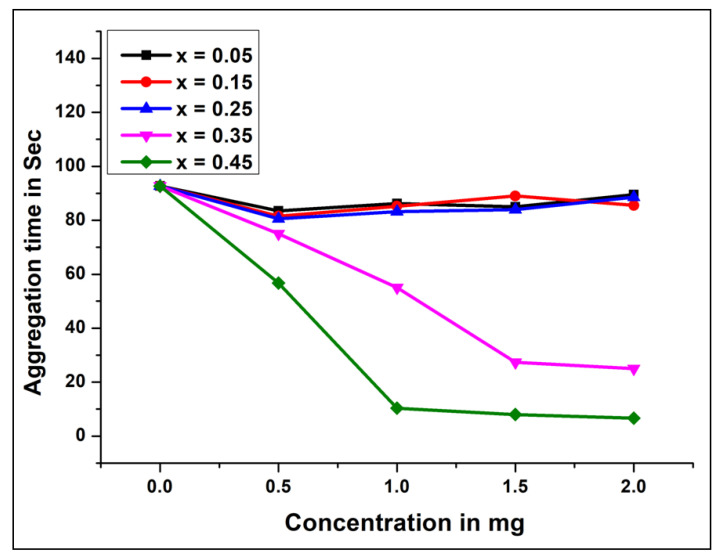
Aggregation time V/s concentration of Ni_(1−x)_Cu_x_Mn_2_O_4_ (x = 0.05, 0.15, 0.25, 0.35, 0.45).

Platelet accumulation was monitored by analyzing light transmission in a CHRONO-LOG Model 700 Aggregometer. Platelet-rich plasma (0.25 mL) was incubated with different concentrations (0–2 mg) of NCB-NPs. Then platelet accumulation was started by adding ADP (10 µM), and aggregation was observed as shown in Figure 5 and Figure 6. All results are mean ± SD of three individual experiments.

To evaluate the effect of NCB-NPs on platelets, platelet aggregation assay was performed in a CHRONO-LOG platelet aggregometer, using PRP with ADP as agonists. The NCB-NPs with x = 0.35 and x = 0.45 inhibited the platelet aggregation to an extent of 80% and 93%, respectively, while remaining inhibited with only 30%. In the general mechanism of platelet aggregation, ADP is released from activated platelets and leads to the self-activation of other platelets, ultimately leading to platelet aggregation. Uncontrolled activation and aggregation of platelets play a crucial role in thrombotic disorders. Thus antiplatelet aggregating agents have therapeutic implications [28,29,30,31]. Anticoagulant properties have been reported from engineered NPs, such as PEG-coated and silver NPs. These anticoagulant and antiplatelet NPs have better application in treating thrombotic disorder [32,33].

#### 2.4.5. Hemolytic Activity

NCB-NPs did not induce hemolysis of the RBCs. Thus, these are devoid of hemolytic activity. The non-hemolytic property indicates the biocompatibility property of the NPs.

## 3. Conclusions

The copper-substituted nickel manganites Ni_(1−x)_Cu_x_Mn_2_O_4_-Bio (NCB-NPs) were produced by using a co-precipitation route at ambient temperature. The synthesized NCB-NPs were characterized by using PXRD, SEM and Raman techniques. XRD characterization confirmed the cubic spinel structure for the synthesized NCB-NPs. The lattice parameter and the crystallite size of the samples was calculated to be 8.3–8.6 Å and 23–28 nm. A significant variation in the lattice parameter and crystallite size has confirmed the doping of copper into the nickel manganites’ structure. A morphological investigation using SEM showed a non-uniform agglomerated polygon and plate-like structure with an increasing size as the copper content increased. The Raman spectral investigation also helped to confirm the cubic spinel structure of NCB-NPs. These NCB-NPs interfered in the blood coagulation cascade showed an anticoagulation effect and also inhibited the platelet aggregation. The hemolytic activity in RBC cells did not show with these NCB-NPs, suggesting their non-toxic nature. In conclusion, this study demonstrated that NCB-NPs can be used in the field of biomedical applications, especially as antithrombotic agents.

## 4. Experimental

### 4.1. Process of Synthesis

The Ni_(1−x)_Cu_x_Mn_2_O_4_ series (x = 0.05, 0.15, 0.25, 0.35, 0.45) (NCB-NPs) were produced by less expensive and effective co-precipitation route (sol–gel). Stoichiometric ratios of AR grade precursors from Burgoyne & Burbridges Company, Mumbai, India (nickel chloride NiCl_2_6H_2_O, manganese chloride MnCl_2_4H_2_O and copper chloride CuCl_2_2H_2_O) were taken without further purification for the preparation. The solution of precursors and ionized water was continuously added with liquid ammonia (NH_3_), with constant stirring until a pH of 7 was obtained. After precipitation and filtration, the wet paste was dried at room temperature for 24 h. Then the dry paste collected was uniformly grinded into a fine powder, with the help of a mortar and pestle. Lastly, the dry powder, using alumina crucible, was sintered at 600 °C in a muffle furnace for four hours to get a dark-colored end-product: nickel copper manganite.

### 4.2. Characterization Techniques

**Figure 7:**
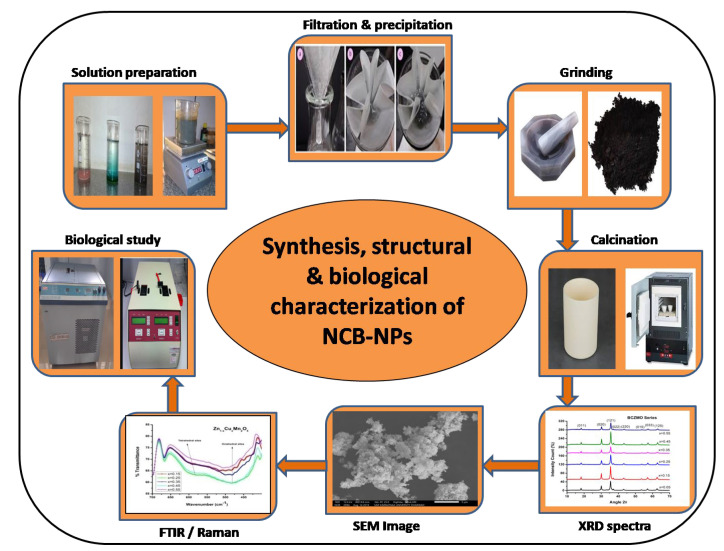
Synthesis and different characterization techniques used for Ni_(1−x)_Cu_x_Mn_2_O_4_ series.

Study of X-ray diffraction of the developed samples was performed with the help of spectra recorded on a Bruker AXS D8 Advance instrument (with X-ray wavelength *λ* = 1.5406 Å, Silicon Lithium PSD detector) (Billerica, MA, USA). The morphology of the samples was determined by the images recorded on SEM (JEOL Model JSM-6390LV, Tokyo, Japan). (XRD and SEM spectra were taken from Sophisticated Test and Instrumentation Centre, Cochin, India.) The Raman spectra were obtained by using Horiba Jobin Vyon, Tokyo, Japan, Model Lab Ram HR from Central Instruments Facility, Indian Institute of Technology Guwahati, Guwahati, India. The flowchart of synthesis and characterizations of NCB-NPs are as shown in Figure 7.

### 4.3. Anticoagulant and Antiplatelet Activity

#### 4.3.1. Plasma Recalcification Time

The plasma coagulant assay was performed in accordance with the method of Quick [34]. Human blood (Registration no.123/PO/C/99/CPCSEA; sanction letter no. SSCPT/IAEC.clear/152A/2016–17 dated 1 October 2016) was collected in 3.2% trisodium citrate in a ratio of 1:9. To get platelet-poor plasma (PPP), the citrated human blood was centrifuged at 37 °C with 1100× *g* for 15 min. Then, PPP (200 µL) was pre-incubated with different concentrations of NCB-NPs for 10 min at 37 °C. Coagulation time was noted down after the addition of 20 micro-liter of 0.25 M CaCl_2_ to the mixture, which was pre-incubated. The time taken for the formation of a visible clot was noted.

#### 4.3.2. Preparation of Platelet-Rich Plasma (PRP) and Platelet-Poor Plasma (PPP)

The preparation of human plasma, one with platelet-rich another with platelet-poor, was performed according to the method of Ardlie and Han [35]. Briefly, the freshly collected blood was centrifuged at ambient temperature at 120× *g* for 15 min to prepare PRP. Similarly, the blood remaining after removal of PRP was centrifuged at 1100× *g* for 15 min to prepare PPP and the platelet count of PRP was adjusted to 4 × 10^8^/mL. The PRP maintained at 37 °C was used within 2 h for the accumulation process. Plastic wares or siliconized glassware were used for all of the abovementioned preparations.

#### 4.3.3. Platelet Aggregation

A Dual-Channel CHRONO-LOG Aggregometer (Model 700), Havertown, PA, USA, was used to monitor platelet aggregation with continuous stirring at 1000 rpm. PRP (250 µL) was pre-incubated with different concentrations of NPs. The platelet aggregation was begun by adding agonists such as ADP and followed the aggregation for 6 min.

#### 4.3.4. Hemolytic Assay

Preparation of erythrocyte suspension: Blood from a healthy individual was collected and mixed with trisodium citrate (3.2%) in the proportion 1:9. The centrifugation of citrated blood was performed at 3000 rpm for 5 min. The supernatant was disposed and the pellet was cleaned three times with normal saline solution (0.9%). The cells were again immersed in normal saline to make a 2% erythrocyte suspension.

#### 4.3.5. Hemolytic Activity

In vitro hemolytic behavior was assayed according to the method of Shin [36]. Different concentrations of NCB-NPs (0–3000 µg) were mixed with 0.5 mL of a 2% erythrocyte suspension, and the reaction volume was made up to 1 mL with normal saline. The mixture was developed at 37 °C for 30 min. After incubation, 2 mL of normal saline was gently put into the mixture, which was further centrifuged at 1500 rpm for 2–3 min. The quantity of hemoglobin left in 1 mL of supernatant was determined at 540 nm. Normal saline and ionized water were taken as minimal and maximal hemolytic controls.

## Figures and Tables

**Table 1:** Crystallite size, dislocation density, grain size and frequencies of absorption bands.

x	Crystallite Size D nm	Dislocation Density f_D_ × 10^15^/m^2^	Grain Size in μm	ν_1_ cm^−1^ (T_h_ Site)	ν_2_ cm^−1^ (O_h_ Site)
0.05	25.03	1.83	2.72	582.42	412.71
0.15	24.15	1.87	2.87	586.28	410.78
0.25	26.38	1.46	3.05	588.21	408.85
0.35	27.15	1.36	3.75	590.14	408.68
0.45	26.18	1.69	4.42	592.06	407.92

ν_1_:Tetrahedral mode; ν_2_: Octahedral mode.
